# Screening of interferon-stimulated genes against avian reovirus infection and mechanistic exploration of the antiviral activity of IFIT5

**DOI:** 10.3389/fmicb.2022.998505

**Published:** 2022-09-15

**Authors:** Sheng Wang, Lijun Wan, Hongyu Ren, Zhixun Xie, Liji Xie, Jiaoling Huang, Xianwen Deng, Zhiqin Xie, Sisi Luo, Meng Li, Tingting Zeng, Yanfang Zhang, Minxiu Zhang

**Affiliations:** Guangxi Key Laboratory of Veterinary Biotechnology, Guangxi Veterinary Research Institute, Nanning, China

**Keywords:** avian reovirus, interferon, interferon-stimulated genes, IFIT5, antiviral response

## Abstract

Avian reovirus (ARV) infection can lead to severe immunosuppression, complications, and secondary diseases, causing immense economic losses to the poultry industry. In-depth study of the mechanism by which the innate immune system combats ARV infection, especially the antiviral effect mediated by interferon, is needed to prevent and contain ARV infection. In this study, ARV strain S1133 was used to artificially infect 7-day-old specific pathogen–free chickens. The results indicated that ARV rapidly proliferated in the immune organs, including the spleen, bursa of Fabricius, and thymus. The viral load peaked early in the infection and led to varying degrees of pathological damage to tissues and organs. Real-time quantitative PCR revealed that the mRNA levels of interferon and multiple interferon-stimulated genes (ISGs) in the spleen, bursa of Fabricius, and thymus were upregulated to varying degrees in the early stage of infection. Among the ISGs, IFIT5, and Mx were the most upregulated in various tissues and organs, suggesting that they are important ISGs for host resistance to ARV infection. Further investigation of the role of IFIT5 in ARV infection showed that overexpression of the IFIT5 gene inhibited ARV replication, whereas inhibition of the endogenously expressed IFIT5 gene by siRNA promoted ARV replication. IFIT5 may be a positive feedback regulator of the innate immune signaling pathways during ARV infection and may induce IFN-α production by promoting the expression of MAD5 and MAVS to exert its antiviral effect. The results of this study help explain the innate immune regulatory mechanism of ARV infection and reveal the important role of IFIT5 in inhibiting ARV replication, which has important theoretical significance and practical application value for the prevention and control of ARV infection.

## Introduction

Avian reovirus (ARV) is an important and prevalent avian pathogen that mainly infects chickens, turkeys, and a few other birds. ARV infection can cause viral arthritis, tenosynovitis, growth retardation, and malabsorption syndrome. It can also lead to severe immunosuppression, which in turn causes vaccine immunization failure and can lead to complications or secondary diseases, causing enormous economic losses to the poultry farming industry (van der Heide, 2000; Dandar et al., 2013; Teng et al., 2014).

The innate immune response is the first line of defense against the virus. After the virus infects the host, the pathogen-related molecular pattern of the virus is specifically recognized by the host’s pattern recognition receptor, and the host senses pathogen invasion, thereby initiating a series of downstream signaling pathways and inducing the production of various antiviral cytokines (Green et al., 2014; Iwasaki and Pillai, 2014). The innate immune response is characterized by extensive function against non-specific antigens and a rapid response. The relationship between ARV and the host innate immune response is a current research area of intense interest. ARV infection can cause significant changes in host innate immune-related pattern recognition receptors and inflammatory cytokines at the transcriptional level, indicating that ARV infection is closely related to recognition by host innate immune-related pattern receptors and the production of inflammatory cytokines (Lin et al., 2010; Lostale-Seijo et al., 2016; Xie et al., 2019, 2020). The interferon (IFN)-mediated antiviral effect is an extremely important link in the host’s natural immune response. IFN itself does not inactivate the virus. After IFN is produced, it binds to the corresponding IFN receptor on cell surfaces and induces the transcriptional expression of many interferon-stimulated genes (ISGs) by activating the JAK-STAT signaling pathway to transmit extracellular signals to the cells (Moraga et al., 2009; Secombes and Zou, 2017).

Interferon-stimulated genes are important antiviral molecules that play an important role in the host’s defense against and elimination of foreign pathogens (Schneider et al., 2014). After reovirus infection in mammals, the expression of many ISGs is activated to resist viral infection (Sherry, 2009; Anafu et al., 2013). As a member of the Reoviridae family, infection by ARV can also induce the expression of IFN and ISGs. Lostale-Seijo et al. (2016) found that ARV infection of chicken embryonic fibroblasts can efficiently induce the expression of IFN-α, IFN-β, Mx, and the double-stranded RNA (dsRNA)-dependent protein kinase (PKR) of the ISG family to exert antiviral effects. Research has also found that the ARV σA protein binds to viral dsRNA in an irreversible manner to inhibit activation of PKR, thereby enabling the virus to resist the antiviral effect of interferon and to defend against or escape the host immune system (Martinez-Costas et al., 2000; Gonzalez-Lopez et al., 2003; Vazquez-Iglesias et al., 2009). In our previous study, we found changes in the mRNA expression patterns of a variety of ISGs in the joints of specific pathogen–free (SPF) chickens infected with ARV S1133; these changes were essentially consistent with the trend of changes in the viral load in the joints, suggesting that IFN-α, IFN-β, and ISGs such as Mx, IFITM3, PKR, OAS, IFIT5, ISG12, Viperin, IFI6, and CD47 play important roles in the defense against ARV invasion, inhibition of ARV replication and proliferation, and viral clearance (Wang et al., 2021).

The immune organs of the body play an important role in maintaining the immune function of the body and preventing the invasion of pathogenic microorganisms. ARV infection can cause damage to tissues and organs such as the spleen, thymus, and bursa of Fabricius in poultry, which is an important cause of immunosuppression from ARV infection (Souza et al., 2018; Crispo et al., 2019). Currently, no report is available on changes in ISG expression in immune organs after ARV infection.

In this study, 7-day-old SPF chickens were artificially infected with ARV strain S1133. By observing pathological changes in the immune organs after ARV infection, we detected the viral load of ARV in different immune tissues and organs, and real-time quantitative PCR was used to evaluate the effects of IFN-related genes and ISGs on expression levels during ARV infection. The immune responses induced by the immune organs after ARV infection were systematically analyzed. ISGs with an important role in ARV infection were screened out, and the antiviral effects of the selected ISGs were verified. The results of this study will further enrich our understanding of the regulatory mechanism of the innate immune response of ARV infection and lay the foundation for investigation of the antiviral molecular mechanism of ISGs.

## Materials and methods

### Ethics statement

This study was approved by the Animal Ethics Committee of Guangxi Veterinary Research Institute. Samples were collected according to protocol #2019C0406 issued by the Animal Ethics Committee of Guangxi Veterinary Research Institute.

### Virus strain

The ARV standard virulent strain S1133 was purchased from the China Institute of Veterinary Drug Control. Before use, ARV was inoculated into the yolk sacs of SPF chicken embryos for proliferation. After the virus was harvested, DF-1 cells were inoculated. Viral titers were determined by the Reed–Muench method.

### Animal experiments

Specific pathogen–free White Leghorn chicken eggs were purchased from Beijing Boehringer Ingelheim Vital Biotechnology Co., Ltd. (Beijing, China) and were hatched using an automatic hatching machine. After hatching, the chicks were transferred to an SPF chicken isolator for rearing. Eighty 7-day-old SPF chickens with good growth were randomly divided into two groups of 40, which were housed in two different isolators. In the ARV infection group, each animal was challenged with 0.1 ml of ARV [10^4^ of the 50% tissue culture infectious dose (TCID_50_)/0.1 ml] by footpad injection. The control group was treated with an equal amount of phosphate-buffered saline (PBS) for footpad injection. After the challenge, the clinical symptoms of the chickens in the ARV infection group and the control group were observed every day. Samples were taken at 1, 2, 3, 4, 5, 6, 7, 10, 14, 21, 28, and 35 days after the challenge. Three chickens were randomly selected as three biological replicates, and the spleen, thymus, and bursa of Fabricius were collected for pathological observation and real-time fluorescence quantitative PCR detection.

### Preparation of pathological sections

The tissue samples collected above were fixed in 10% neutral formalin, and tissue sections were prepared by Shuiyuntian Biotechnology Co., Ltd. (Guangzhou, China). The sections were stained with hematoxylin–eosin to observe pathological changes in the spleen, bursa of Fabricius, and thymus after ARV infection.

### RNA extraction and cDNA synthesis

RNA was extracted from collected tissue samples using the RNA purification kit GeneJET RNA Purification Kit (Thermo Scientific, USA). cDNA was synthesized by reverse transcription using the MaximaTM H Minus cDNA Synthesis Master Mix with dsDNase (Thermo Scientific) and stored at −80°C until real-time fluorescence quantitative PCR.

### Real-time fluorescence quantitative PCR

Based on the gene sequence information in GenBank, primers for the ARV σC gene, IFN, and ISGs were synthesized by Invitrogen (Table 1). GAPDH was used as the internal reference gene for real-time fluorescence quantitative PCR. Real-time fluorescence quantitative PCR was performed using PowerUp SYBR Green Master Mix (Thermo Scientific) and a QuantStudio 5 real-time PCR system (Thermo Life Tech ABI, USA). Gene expression was compared by the 2^−ΔΔ*C*t^ method.

**TABLE 1:** Primers used in this study.

Gene	GenBank accession number	Primer sequences (5′-3′)
ARV σC	L39002.1	F:CCACGGGAAATCTCACGGTCACT,
		R:TACGCACGGTCAAGGAACGAATGT
IFN-α	AB021154.1	F:ATGCCACCTTCTCTCACGAC,
		R: AGGCGCTGTAATCGTTGTCT
IFN-β	X92479.1	F:ACCAGGATGCCAACTTCT,
		R:TCACTGGGTGTTGAGACG
OAS	NM_205041.1	F:GCGGTGAAGCAGACGGTGAA,
		R:CGATGATGGCGAGGATGTG
IFITM3	KC876032.1	F:GGAGTCCCACCGTATGAAC,
		R:GGCGTCTCCACCGTCACCA
MX	AY695797.1	F:AACGCTGCTCAGGTCAGAAT,
		R:GTGAAGCACATCCAAAAGCA
PKR	AB125660.1	F:CCTCTGCTGGCCTTACTGTCA,
		R:AAGAGAGGCAGAAGGAATAATTTGCC
Viperin	EU427332.1	F:CAGTGGTGCCGAGATTATGC,
		R:CACAGGATTGAGTGCCTTGA
IFIT5	XM_421662	F:CTCCCAAATCCCTCTCAACA,
		R:AAGCAAACGCACAATCATCA
ISG12	BN000222.1	F:TCCTCAGCCATGAATCCGAACA,
		R:GGCAGCCGTGAAGCCCAT
ZFP313	AY604724.1	F:ATCGCTTTACCTTTCCTTG,
		R:GTGCCATCGTATCATCTTCA
IFI6	NM_001001296.5	F:CACTCCTCAGGCTTTACC,
		R:GACCGATGCTTCTTTCTATT
MDA5	NM_001193638	F:CAGCCAGTTGCCCTCGCCTCA,
		R: AACAGCTCCCTTGCACCGTCT
LGP2	MF563595.1	F:CCAGAATGAGCAGCAGGAC,
		R:AATGTTGCACTCAGGGATGT
MAVS	MF289560.1	F:CCTGACTCAAACAAGGGAAG,
		R:AATCAGAGCGATGCCAACAG
TRAF3	XM_040672281.1	F:GGACGCACTTGTCGCTGTTT,
		R:CGGACCCTGATCCATTAGCAT
TRAF6	XM_040673314.1	F:GATGGAGACGCAAAACACTCAC,
		R:GCATCACAACAGGTCTCTCTTC
IKKε	XM_428036.4	F:TGGATGGGATGGTGTCTGAAC,
		R:TGCGGAACTGCTTGTAGATG
TBK1	MF159109.1	F:AAGAAGGCACACATCCGAGA,
		R:GGTAGCGTGCAAATACAGC
IRF7	NM_205372.1	F:CAGTGCTTCTCCAGCACAAA,
		R:TGCATGTGGTATTGCTCGAT
NF-κB	NM_205129.1	F:CATTGCCAGCATGGCTACTAT,
		R:TTCCAGTTCCCGTTTCTTCAC
GAPDH	NM_204305.1	F:GCACTGTCAAGGCTGAGAACG,
		R:GATGATAACACGCTTAGCACCAC

### Overexpression of the IFIT5 protein

With reference to the chicken IFIT5 gene sequence (accession number: NM_001320422.1), the IFIT5 recombinant plasmid pEF1α-Myc-IFIT5 was constructed. DF-1 cells were cultured in six-well plates overnight, and 2 μl Lipofectamine™ 3000 (Invitrogen, USA) was used to transfect 1 μg of pEF1α-Myc-IFIT5 plasmid into DF-1 cells to overexpress the IFIT5 protein. At 24 h after transfection, cells were infected with ARV strain S1133 at a multiplicity of infection (MOI) of 1. Cell samples and culture supernatants were collected 24 h after infection. RNA was extracted from the collected cell samples, and cDNA was synthesized. Real-time fluorescence quantitative PCR was run to measure ARV replication and changes in the expression of molecules related to the innate immune signaling pathways. The primer sequences of the ARV σC gene and molecules related to natural immune signaling are shown in Table 1 and were synthesized by Invitrogen. In addition, the culture supernatant was infected with DF-1 cells, and the viral titer was determined by the Reed–Muench method to measure ARV replication.

### IFIT5 RNA interference assay

Three small interfering RNAs (siRNAs) targeting the IFIT5 gene were designed and synthesized by GenePharma (Table 2). DF-1 cells were cultured in six-well plates overnight, and 2 μl of Lipofectamine RNAiMAX (Invitrogen) was used to transfect 1 μg of 200 nM IFIT5 of each siRNA separately or the negative control (siNC) to inhibit IFIT5 protein expression. Cell samples and culture supernatants were collected 24 h after transfection and infected with ARV strain S1133 (MOI = 1). We extracted RNA from the cells to detect ARV replication and changes in the expression of molecules related to innate immune signaling pathways. Viral replication was measured in the culture supernatant.

**TABLE 2:** siRNA sequences targeting the IFIT5 gene.

siRNA	Sequences (5′-3′)	Sequences (3′-5′)
si225	GGAAGAAUCAAUAGAGGAUTT	AUCCUCUAUUGAUUCUUCCTT
si738	GCGUGCACUGAAACUGAAUTT	AUUCAGUUUCAGUGCACGCTT
si1453	GGUGAGAAGCUUGAAGCAATT	UUGCUUCAAGCUUCUCACCTT
siNC	UUCUCCGAACGUGUCACGUTT	ACGUGACACGUUCGGAGAATT

### Statistical analysis

All data were plotted in GraphPad Prism 8 software. The results are expressed as the mean ± standard deviation (SD). Statistical analysis was performed using IBM SPSS Statistics 2.0 software. Student’s t-test was used to evaluate differences between groups. *P* < 0.05 indicated statistically significant differences, which are marked with ^∗^ in the figures; *P* < 0.01 indicated very significant differences, which are marked with ^∗∗^ in the figures.

## Results

### Pathological changes in immune organs after avian reovirus infection

**FIGURE 1:**
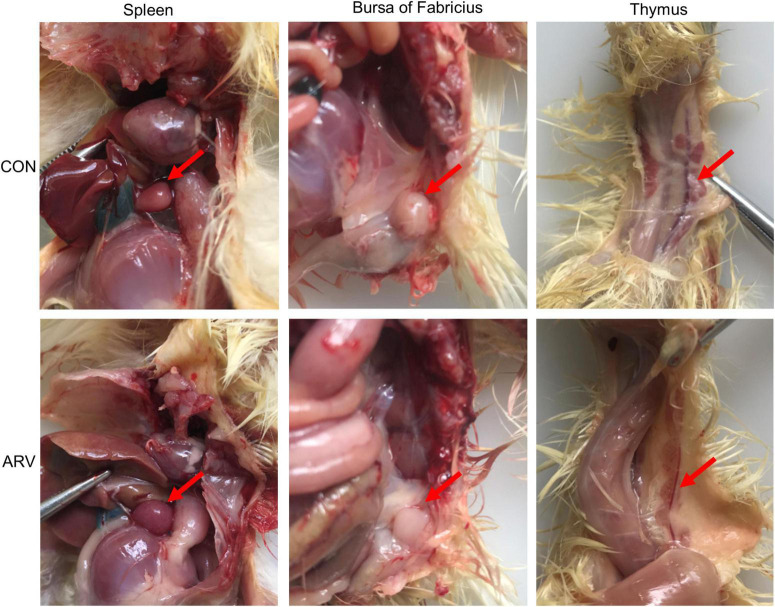
Pathological changes in the spleen, bursa of Fabricius, and thymus in SPF chickens infected with ARV. In the control group (CON), SPF chickens were injected with 0.1 ml of PBS into the footpad. In the experimental group (ARV), ARV strain S1133 (10^4^ TCID_50_/0.1 ml) was injected into SPF chickens through the footpad. After ARV infection of SPF chickens, dissection results revealed edema in the spleen and atrophy in the bursa of Fabricius and thymus.

**FIGURE 2:**
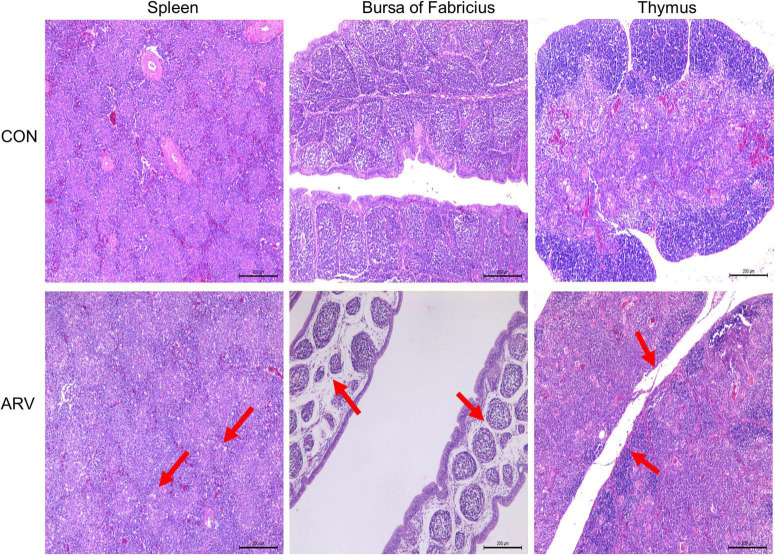
Histopathological changes in the spleen, bursa of Fabricius, and thymus in SPF chickens infected with ARV. In the control group (CON), SPF chickens were injected with 0.1 ml of PBS into the footpad. In the experimental group (ARV), ARV strain S1133 (10^4^ TCID_50_/0.1 ml) was injected into SPF chickens through the footpad. The arrow in the spleen indicates extensive lymphocyte degeneration and necrosis. Arrows in the bursa of Fabricius indicate lymphatic follicular structural loosening, lymphocytic degeneration and necrosis, heterophilic cell infiltration, interstitial loosening, and inflammatory cell infiltration. Arrows in the thymus indicate atrophy, reduced cortex/medulla proportions, and decreased cortical lymphocytes.

After ARV infection of SPF chickens, dissection results revealed edema in the spleen and atrophy of the bursa of Fabricius and thymus (Figure 1). Histopathology indicated that the three immune organs all had typical histopathological changes. The spleen mainly showed extensive lymphocyte degeneration and necrosis. The bursa of Fabricius showed a loose structure of lymphoid follicles, lymphocyte degeneration and necrosis, heterophilic cell infiltration, interstitial porosity, and inflammatory cell infiltration, as well as local lymphoid follicular atrophy, interstitial widening, and fibrosis. The thymus showed atrophy, smaller cortex/medulla proportions, and few cortical lymphocytes (Figure 2).

#### Viral load in immune organs after avian reovirus infection

**FIGURE 3:**
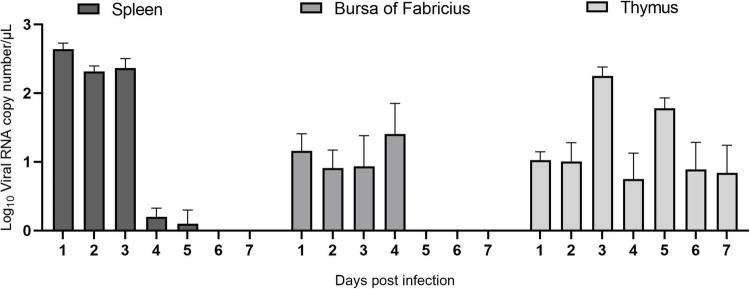
The ARV copy number in the spleen, bursa of Fabricius, and thymus in ARV-infected SPF chickens. ARV strain S1133 (10^4^ TCID_50_/0.1 ml) was used to infect chickens through the footpad. At 1, 2, 3, 4, 5, 6, 7, 10, 14, 21, 28, and 35 days postinfection (dpi), three chickens were randomly collected from each group, and the ARV viral load was measured by real-time quantitative PCR. The data are the mean ± SD of three independent experiments.

To study ARV proliferation in the immune organs, the ARV viral load in the above tissues and organs was measured by real-time quantitative PCR. The results are shown in Figure 3. After ARV infection, ARV in the spleen began to proliferate rapidly at 1 day postinfection (dpi) until reaching the peak level, remained at a high level between 2 and 3 dpi, suddenly decreased at 4 dpi, and was not detectable after 6 dpi. ARV was detected only between 1 and 4 dpi in the bursa of Fabricius, but the viral copy number was low. ARV was detected in the thymus at 1 dpi, peaked at 3 dpi, and was undetectable after 10 dpi. Throughout the experiment, the ARV σC gene was detected by real-time quantitative PCR in all control samples, and the results were all negative.

### Transcription level of IFN in immune organs after avian reovirus infection

**FIGURE 4:**
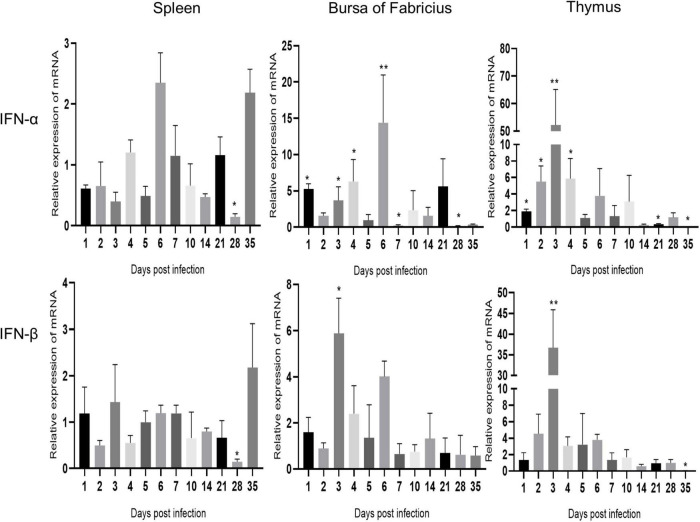
Changes in IFN transcription levels in the spleen, bursa of Fabricius, and thymus after ARV infection. ARV strain S1133 (10^4^ TCID_50_/0.1 ml) was used to infect chickens through the footpad. At 1, 2, 3, 4, 5, 6, 7, 10, 14, 21, 28, and 35 days after infection (dpi), three chickens were randomly collected from each group, and changes in IFN gene expression were analyzed using real-time quantitative PCR. The data are the mean ± SD of three independent experiments. Asterisks indicate significant differences (*P < 0.05, **P < 0.01).

As shown in Figure 4, IFN-α and IFN-β showed similar changes in the same immune organ after ARV infection. The expression levels of IFN-α and IFN-β in the spleen did not change significantly and were significantly downregulated only at 28 dpi (*P* < 0.05). The mRNA levels of IFN-α and IFN-β in the bursa of Fabricius were both upregulated between 1 and 6 dpi, and the expression level of IFN-α was significantly higher than that of IFN-β. IFN-α peaked at 6 dpi at 14.39 times the expression level in the control group (*P* < 0.01), and IFN-β peaked at 3 dpi at 5.89 times the control level (*P* < 0.05). The mRNA levels of IFN-α and IFN-β in the thymus were significantly upregulated between 1 and 10 dpi, and both peaked at 3 dpi. The IFN-α level was 52.25 times that in the control group (*P* < 0.01), and the IFN-β level was 36.67 times that in the control group (*P* < 0.01).

### Expression levels of interferon-stimulated genes mRNAs in immune organs after avian reovirus infection

**FIGURE 5:**
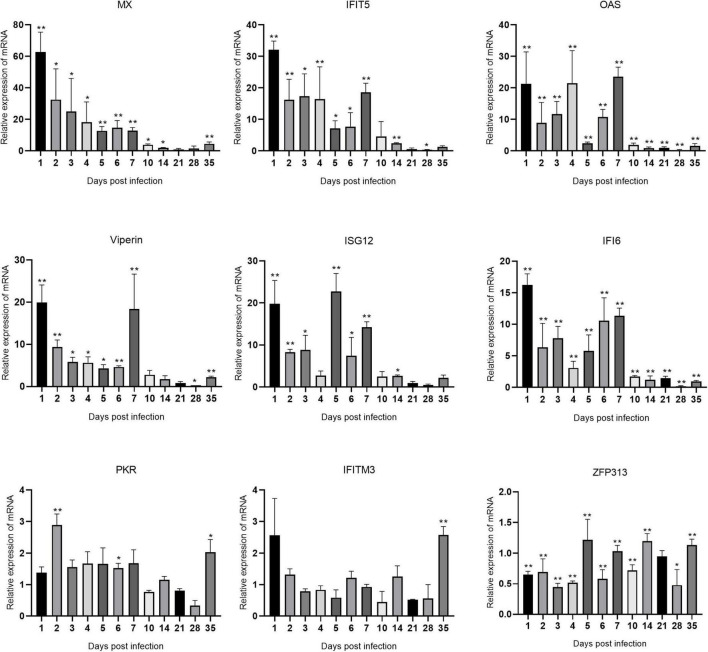
Changes in ISG transcription levels in the spleen after ARV infection. ARV strain S1133 (10^4^ TCID_50_/0.1 ml) was used to infect chickens through the footpad. At 1, 2, 3, 4, 5, 6, 7, 10, 14, 21, 28, and 35 days after infection (dpi), three chickens were randomly collected from each group, and changes in ISG mRNA levels in the spleen were measured by real-time quantitative PCR. The data are the mean ± SD of three independent experiments. Asterisks indicate significant differences (*P < 0.05, **P < 0.01).

**FIGURE 6:**
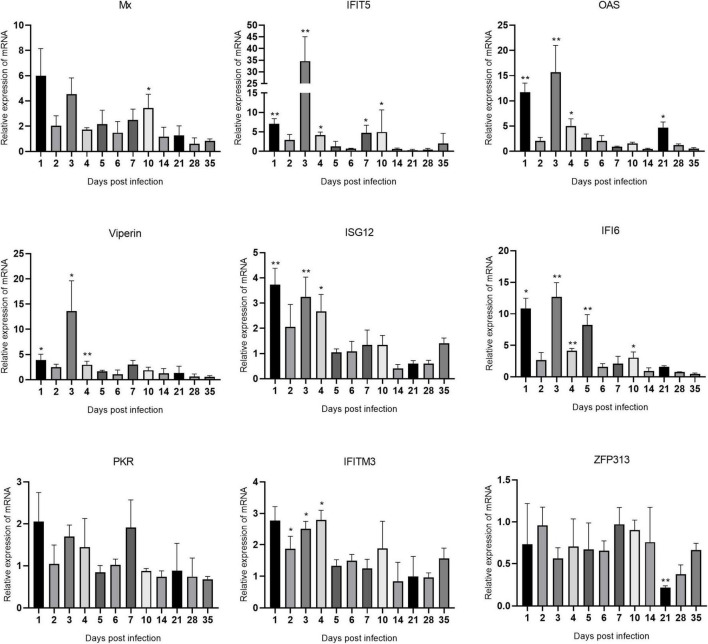
Changes in ISG transcription levels in the bursa of Fabricius after ARV infection. ARV strain S1133 (10^4^ TCID_50_/0.1 ml) was used to infect chickens through the footpad. At 1, 2, 3, 4, 5, 6, 7, 10, 14, 21, 28, and 35 days after infection (dpi), three chickens were randomly collected from each group, and changes in ISG mRNA levels in the bursa of Fabricius were measured by real-time quantitative PCR. The data are the mean ± SD of three independent experiments. Asterisks indicate significant differences (*P < 0.05, **P < 0.01).

**FIGURE 7:**
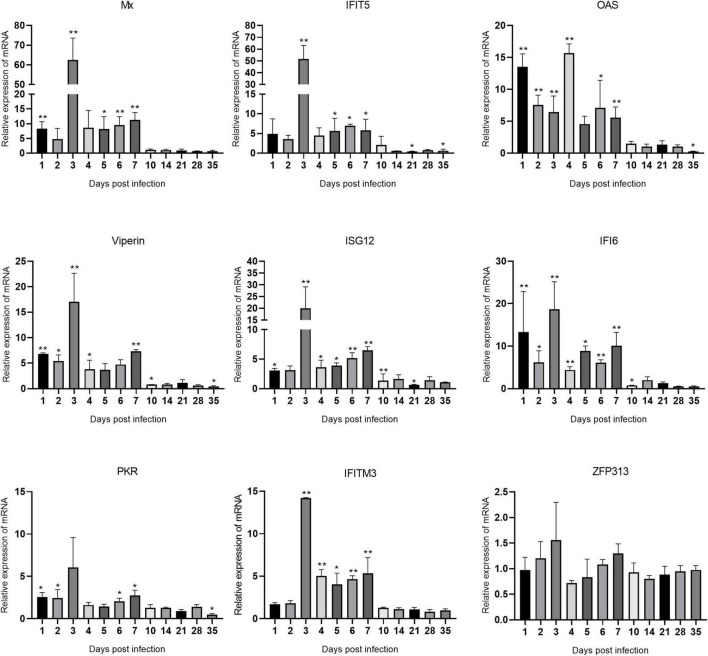
Changes in ISG transcription levels in the thymus after ARV infection. ARV strain S1133 (10^4^ TCID_50_/0.1 ml) was used to infect chickens through the footpad. At 1, 2, 3, 4, 5, 6, 7, 10, 14, 21, 28, and 35 days after infection (dpi), three chickens were randomly collected from each group, and changes in ISG mRNA levels in the thymus were measured by real-time quantitative PCR. The data are the mean ± SD of three independent experiments. Asterisks indicate significant differences (*P < 0.05, **P < 0.01).

The changes in the expression of ISGs in the spleen, bursa of Fabricius, and thymus are shown in Figures 5–7 and Supplementary Table 1. ARV infection rapidly and significantly upregulated nine common avian ISGs in immune organs at the mRNA level to different degrees.

The changes in the mRNA levels of ISGs in the spleen after ARV infection are shown in Figure 5. The most significant increases were observed in Mx and IFIT5. Mx, IFIT5, IFI6, and Viperin were rapidly upregulated and reached their peak values at 1 dpi, which were 62.85, 32.11, 16.21, and 19.95 times the corresponding control levels, respectively (all *P* < 0.01). OAS and ISG12 were also rapidly and significantly upregulated at 1 dpi. OAS peaked at 7 dpi at 23.51 times the control level, and ISG12 peaked at 5 dpi at 22.74 times the control level (both *P* < 0.01). ZFP313 was downregulated between 1 and 4 dpi (*P* < 0.01) and then increased to a level not significantly different from that in the control group. PKR and IFITM3 did not significantly change over time and were not significantly different from the control levels at most time points.

The changes in the mRNA levels of ISGs in the bursa of Fabricius are shown in Figure 6. The increase in IFIT5 was the most significant. The changes in IFIT5, OAS, Viperin, and IFI6 were similar, which were rapidly upregulated at 1 dpi, decreased at 2 dpi, and peaked at 3 dpi at 34.61, 15.69, 13.64, and 12.67 times the corresponding control levels, respectively (*P* < 0.01 or *P* < 0.05). ISG12, IFITM3, Mx, and PKR were also slightly upregulated after infection. ZFP313 was slightly downregulated throughout the experiment.

Changes in the mRNA levels of ISGs in the thymus are shown in Figure 7. Mx and IFIT5 showed the most significant increases. Mx, IFIT5, Viperin, ISG12, IFI6, PKR, and IFITM3 exhibited similar changes in transcription levels, which were all rapidly upregulated at 1 dpi, peaked at 3 dpi, and remained high between 1 and 7 dpi. Mx and IFIT5 were the most upregulated at 62.57 and 51.57 times the control levels, respectively (both *P* < 0.01). Viperin, ISG12, IFI6, and IFITM3 at 3 dpi were upregulated to 17.01, 19.96, 18.68, and 14.19 times the control levels, respectively (all *P* < 0.01). OAS was rapidly upregulated at 1 dpi after infection and peaked at 4 dpi at 15.69 times the control level (*P* < 0.01). PKR was slightly upregulated after infection. The expression level of ZFP313 did not change significantly during the infection process. Among the many upregulated ISGs, IFIT5 and Mx had the most significantly upregulated mRNA levels in these three tissues and organs, indicating that IFIT5 and Mx are important host ISGs against ARV infection.

### High IFIT5 expression reduced avian reovirus replication

**FIGURE 8:**
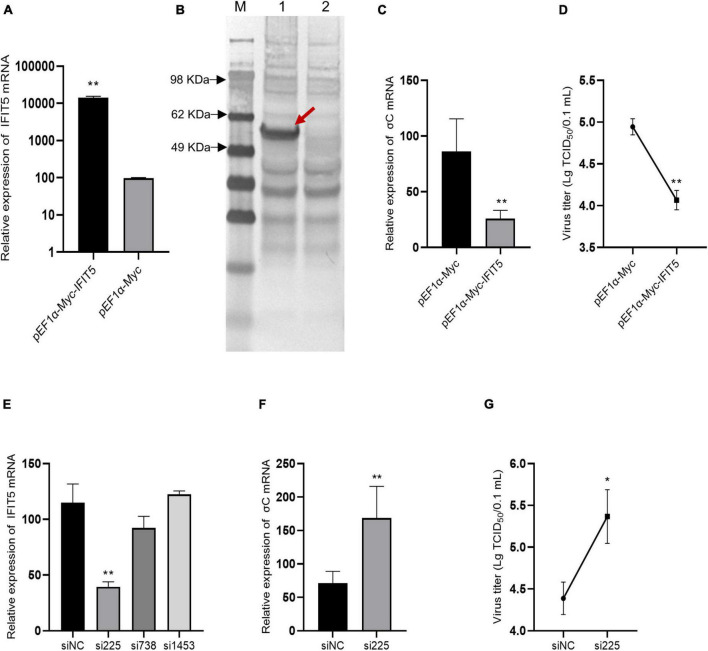
IFIT5 inhibits ARV replication in DF-1 cells. DF-1 cells were transfected with pEF1α-Myc-IFIT5 or pEF1α-Myc plasmids. Both real-time quantitative PCR **(A)** and Western blotting **(B)** confirmed that IFIT5 was highly expressed in DF-1 cells. DF-1 cells were transfected with pEF1α-Myc-IFIT5 or pEF1α-Myc plasmid and infected with ARV strain S1133 (MOI = 1). Viral replication was measured by real-time quantitative PCR **(C)** and viral titers **(D)** at 24 h after infection. The inhibitory efficiency of three IFIT5 siRNAs was compared by real-time quantitative PCR **(E)**. DF-1 cells were transfected with si225 or siNC and infected with ARV strain S1133 (MOI = 1). Viral replication was measured by real-time quantitative PCR **(F)** and viral titers **(G)** at 24 h after infection. The data are the mean ± SD of three independent experiments. Asterisks indicate significant differences (*P < 0.05, **P < 0.01).

DF-1 cells were transfected with the pEF1α-Myc-IFIT5 recombinant plasmid. Real-time quantitative PCR results revealed that IFIT5 gene expression was significantly upregulated after transfection with the pEF1α-Myc-IFIT5 recombinant plasmid. Western blot detection using the Myc-tagged antibody indicated that the IFIT5 protein (approximately 56 kDa) was normally expressed in DF-1 cells transfected with the pEF1α-Myc-IFIT5 recombinant plasmid (Figures 8A,B). After IFIT5 overexpression, cells were infected with ARV. Real-time quantitative PCR was used to measure the mRNA level of the ARV σC gene. The results showed that the mRNA level of the ARV σC gene was significantly reduced after IFIT5 overexpression (Figure 8C). The measurement results for ARV titers in the cell supernatant also indicated that the viral titer of the cell supernatant was significantly reduced after IFIT5 overexpression (Figure 8D).

Therefore, we speculated that IFIT5 could negatively regulate ARV replication and that downregulation of IFIT5 expression could promote ARV replication. We designed and synthesized three siRNAs targeting IFIT5, which were transfected into DF-1 cells to inhibit the expression of endogenous IFIT5. The results revealed that si225 had the best inhibitory effect (Figure 8E). DF-1 cells were transfected with si225 and siNC and inoculated with ARV at 24 h after transfection. ARV replication was detected based on gene expression and viral titers. The results aligned with our expectations (Figures 8F,G). Inhibition of IFIT5 expression promoted ARV replication.

### IFIT5 acts as a positive feedback regulator in natural immune signaling pathways

**FIGURE 9:**
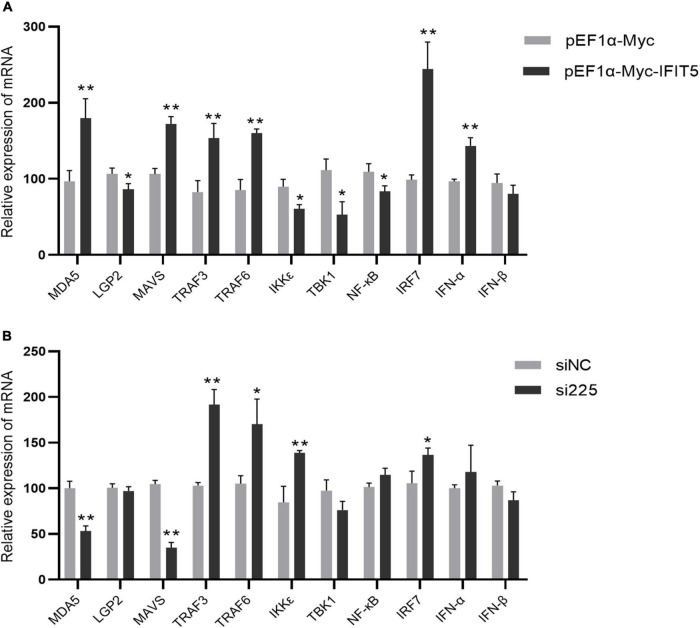
IFIT5 acts as a positive feedback regulator in natural immune signaling pathways. DF-1 cells were transfected with pEF1α-Myc-IFIT5 or pEF1α-Myc plasmids and infected with the ARV strain S1133 (MOI = 1). Expression changes in genes related to the innate immune signaling pathway were measured by real-time quantitative PCR (A) at 24 h after infection. DF-1 cells were transfected with si225 or siNC and infected with ARV strain S1133 (MOI = 1). Expression changes in genes related to the innate immune signaling pathway were measured by real-time quantitative PCR (B) at 24 h after infection. The data are the mean ± SD of three independent experiments. Asterisks indicate significant differences (*P < 0.05, **P < 0.01).

The above results indicate that IFIT5 can inhibit ARV proliferation, but the specific antiviral mechanism is still unclear. We overexpressed or knocked down IFIT5 in DF-1 cells and infected them with ARV to analyze the effect of IFIT5 on the expression of innate immune signaling pathway–related factors during ARV infection by real-time quantitative PCR. The results are shown in Figure 9 and Supplementary Table 2. The mRNAs encoding MAD5 and MAVS were significantly upregulated after IFIT5 overexpression and significantly downregulated after IFIT5 knockdown. MAD5 and MAVS were upregulated 1.85- and 1.62-fold after IFIT5 overexpression, respectively, and MDA5 and MAVS were downregulated 0.52- and 0.33-fold after IFIT5 knockdown, respectively (*P* < 0.01). The mRNA levels of IRF7, TRAF3, and TRAF6 were significantly upregulated after IFIT5 overexpression or knockdown (*P* < 0.05 or *P* < 0.01). The mRNA levels of LGP2, TBK1, and NF-κB were significantly downregulated after IFIT5 overexpression (*P* < 0.05), but no significant difference was observed after IFIT5 knockdown. The mRNA expression level of IKKε was significantly downregulated after IFIT5 overexpression and was significantly upregulated after IFIT5 knockdown (*P* < 0.05 or *P* < 0.01). The mRNA encoding IFN-α was significantly upregulated after IFIT5 overexpression at 1.48 times the control levels (*P* < 0.01), but no significant difference was noted after IFIT5 inhibition. No significant difference in the mRNA expression level of IFN-β after overexpression or inhibition of IFIT5 was identified. These results suggest that IFIT5 may be a positive feedback regulator of innate immune signaling pathways. IFIT5 may promote the expression of MAD5 and MAVS, thereby inducing IFN-α production to exert an antiviral effect.

## Discussion

Avian reovirus is an important avian immunosuppressive pathogen, and innate immunity is the first line of defense against the virus (Van de Zande and Kuhn, 2007). Therefore, in-depth study of the innate immune response induced in immune organs after ARV infection will be valuable to reveal the pathogenic mechanism of ARV and the antiviral mechanism of the host. This study analyzed the effect of ARV infection on immune organs. Dissection results revealed that the spleen was swollen in the early stage of ARV infection, while the thymus and the bursa of Fabricius showed atrophy. Pathology indicated significant histopathological changes in the spleen, bursa of Fabricius, and thymus in the early stage of ARV infection, and these symptoms were gradually relieved in the late stage of infection, which is consistent with previous studies (Lin et al., 2007). We further measured the ARV viral loads in the spleen, bursa of Fabricius, and thymus by real-time quantitative PCR. The results showed that ARV rapidly proliferated in several tissues and organs, the viral load peaked in the early stage of infection, and the pattern of change in the viral load was consistent with the pattern of histopathological changes in the spleen, bursa of Fabricius, and thymus, i.e., high proliferation of ARV leads to pathological damage, and this pathological damage gradually recovers when ARV is eradicated from the tissues in the later stages of infection. This finding indicates that the critical period of interaction of ARVs and hosts is mainly the early stage of infection, and ARV proliferation in the host is closely related to pathological damage. In addition, the viral load in the spleen was significantly higher than those in the bursa of Fabricius and the thymus, suggesting that the spleen is the main immune organ attacked by ARV.

Interferon is an important cytokine. IFN-α and IFN-β are type I interferons. After IFN is synthesized, it mainly exerts direct antiviral effects by inducing a series of ISGs. IFN and ISGs play important roles in the antiviral infection process of the body (Schoggins et al., 2011; Tarradas et al., 2014). ARV infection can induce upregulation of IFNs in chicken embryonic fibroblasts (Lostale-Seijo et al., 2016). In this study, real-time quantitative PCR revealed differences in the expression levels of and changes in IFN-α and IFN-β in different tissues and organs after ARV infection. The mRNA levels of IFN-α and IFN-β were differentially upregulated in both the bursa of Fabricius and thymus at the early stage of ARV infection. IFN-α expression was significantly higher than IFN-β expression in the bursa of Fabricius. Both IFN-α and IFN-β were significantly upregulated in the thymus, while IFN-α and IFN-β were not significantly upregulated in the spleen. Therefore, ARV infection is closely related to IFN expression, but the antiviral mode of attack of different tissues and organs may differ after ARV infection. We also measured changes in the expression of ISGs after ARV infection by quantitative PCR. The expression levels of various ISGs in the spleen, bursa of Fabricius, and thymus showed changes at the transcriptional level; that is, all genes were rapidly upregulated at the early stage of infection and reached maximum expression at 1 to 5 days after infection. This study found similar results as previous studies. In the peripheral blood lymphocytes of ARV-infected SPF chickens, type I IFN (IFN-α, IFN-β), type II IFN (IFN-γ), and ISGs (Mx, IFITM1, and OAS) were all upregulated in the early stage of infection, with their expression peaking at 3 days after infection (Xie et al., 2019). In our study, the changes in the mRNA levels of ISGs were essentially consistent with the changes in the viral load levels in tissues and organs. That is, when the ARV copy number increased, the mRNA levels of ISGs increased correspondingly, and after the proliferation of ARV was inhibited, the mRNA expression of ISGs also decreased correspondingly, which was associated with the dynamic pathological changes in tissues and organs. We speculate that ISGs play an important role in resisting ARV invasion, inhibiting ARV replication and proliferation, and promoting viral clearance. We found that the mRNA level of IFN in the spleen did not change significantly, but a variety of ISGs in the spleen were significantly upregulated, suggesting that the spleen might regulate the expression of ISGs in an IFN-independent manner. We also found some differences in the types and expression levels of ISGs regulated in different tissues and organs after ARV infection. Among the many upregulated ISGs, IFIT5 and Mx were the most highly upregulated in all three tissues: 62.85- (Mx) and 32.11-fold (IFIT5) in the spleen; 34.61-fold (IFIT5) in the bursa of Fabricius; and 62.57- (Mx) and 51.57-fold (IFIT5) in the thymus, respectively. In another study, we also found that in joints and target organs after ARV infection, Mx and IFIT5 increased the most significantly among the many ISGs, with the highest upregulation degrees of 748.05-fold for Mx and 660.88-fold for IFIT5, indicating that they are two important ISGs in host resistance to ARV infection (Wang et al., 2021). Lostale-Seijo et al. (2016) found that ARV can effectively induce Mx expression and exert antiviral effects after in vitro infection of chicken embryonic fibroblasts. No other study has reported on the role of IFIT5 in ARV infection and its mechanism.

The IFIT family contains important ISGs. To date, four typical IFIT family members have been identified in the human genome, namely, IFIT1, IFIT2, IFIT3, and IFIT5. IFIT family genes exist in different numbers in different species, and only IFIT5 exists in the avian genome. IFIT5 can be upregulated after stimulation by various viruses, poly(I:C), IFN-α, and IFN-β (Vladimer et al., 2014; Santhakumar et al., 2018). IFIT5 is strongly induced by RNA viruses and much more weakly induced by DNA virus infection (Zhang et al., 2013; Zhang et al., 2021). A variety of avian viruses also upregulate IFIT5 expression. Avian-derived IFIT5 has a broad-spectrum antiviral effect (Matulova et al., 2013; Liu et al., 2015; Wang et al., 2015; Li et al., 2020; Wu et al., 2020). Our study indicated that IFIT5 overexpression in DF-1 cells could inhibit ARV replication, whereas knockdown of endogenous IFIT5 expression in DF-1 cells by siRNA promoted ARV replication, indicating that IFIT5 genes are important ISGs for the host to resist ARV infection. IFIT5 has various antiviral mechanisms, as confirmed in mammals. IFIT5 can positively regulate type I IFNs to further exert its antiviral function (Zhang et al., 2013, Zheng et al., 2015; Li et al., 2020). IFIT5 can negatively regulate the type I IFN signaling pathway (Zhang et al., 2021). However, studies on poultry-derived IFIT5 are limited, and its mechanism of action still requires further study. In this study, real-time quantitative PCR was used to measure the effect of IFIT5 expression on the expression of factors related to the innate immune signaling pathway during ARV infection and found that IFIT5 overexpression significantly upregulated the mRNA levels of IFN-α to 1.48 times the control levels, but no significant difference was found after knockdown of IFIT5. In addition, IFIT5 overexpression significantly upregulated the mRNA levels of MAD5 and MAVS by 1.85- and 1.62-fold, respectively, while knockdown of IFIT5 significantly downregulated them by 0.52- and 0.33-fold, respectively. We speculate that IFIT5 is a positive feedback regulator of the innate immune signaling pathway during ARV infection. IFIT5 may exert its antiviral effects by promoting the expression of MAD5 and MAVS, which in turn induces IFN-α production. In this study, a dynamic analysis of ISGs in the spleen, bursa of Fabricius, and thymus after ARV infection was performed. We identified IFIT5 as a key antiviral candidate gene during ARV infection and conducted preliminary studies on the role of IFIT5 in the process of ARV infection. These research results provide new insights into the interaction between ARVs and hosts, which will facilitate the prevention and control of ARVs. The specific regulatory mechanism of IFIT5 during ARV infection and the mechanisms by which ARV antagonizes the antiviral effects of IFIT5 still require further research.

## Data availability statement

The original contributions presented in this study are included in the article/Supplementary material, further inquiries can be directed to the corresponding author.

## Ethics statement

The animal study was reviewed and approved by the Animal Ethics Committee of Guangxi Veterinary Research Institute, 2019 C0406.

## Author contributions

ZXX designed and coordinated the study and helped to review the manuscript. SW performed the experiments, analyzed the data, and wrote the manuscript. LW and HR assisted in completing the experiment and revised the manuscript. LX, JH, XD, ZQX, SL, ML, TZ, YZ, and MZ assisted with the animal experiments. All authors contributed to the article and approved the submitted version.

## Supplementary material

Supplementary Table 1 Supplementary data-values for each animal.

Supplementary Table 2 Supplementary data-innate immune signaling pathways.
